# Effect of Negative Online Reviews and Physician Responses on Health Consumers’ Choice: Experimental Study

**DOI:** 10.2196/46713

**Published:** 2024-03-12

**Authors:** Xi Han, Yongxi Lin, Wenting Han, Ke Liao, Kefu Mei

**Affiliations:** 1 School of Business Administration Guangdong University of Finance & Economics Guangzhou China; 2 School of Management Science and Engineering Shandong University of Finance and Economics Jinan China; 3 Department of Internal Neurology Xiangyang Central Hospital Xiangyang China

**Keywords:** negative review, proportion, claim type, attribution theory, physician-rating websites, consumer, physician response

## Abstract

**Background:**

The COVID-19 pandemic has highlighted the importance of online medical services. Although some researchers have investigated how numerical ratings affect consumer choice, limited studies have focused on the effect of negative reviews that most concern physicians.

**Objective:**

This study aimed to investigate how negative review features, including proportion (low/high), claim type (evaluative/factual), and physician response (absence/presence), influence consumers’ physician evaluation process under conditions in which a physician’s overall rating is high.

**Methods:**

Using a 2×2×2 between-subject decision-controlled experiment, this study examined participants’ judgment on physicians with different textual reviews. Collected data were analyzed using the t test and partial least squares–structural equation modeling.

**Results:**

Negative reviews decreased consumers’ physician selection intention. The negative review proportion (β=–0.371, *P*<.001) and claim type (β=–0.343, *P*<.001) had a greater effect on consumers’ physician selection intention compared to the physician response (β=0.194, *P*<.001). A high negative review proportion, factual negative reviews, and the absence of a physician response significantly reduced consumers’ physician selection intention compared to their counterparts. Consumers’ locus attributions on the negative reviews affected their evaluation process. Physician attribution mediated the effects of review proportion (β=–0.150, *P*<.001), review claim type (β=–0.068, *P*=.01), and physician response (β=0.167, *P*<.001) on consumer choice. Reviewer attribution also mediated the effects of review proportion (β=–0.071, *P*<.001), review claim type (β=–0.025, *P*=.01), and physician response (β=0.096, *P*<.001) on consumer choice. The moderating effects of the physician response on the relationship between review proportion and physician attribution (β=–0.185, *P*<.001), review proportion and reviewer attribution (β=–0.110, *P*<.001), claim type and physician attribution (β=–0.123, *P*=.003), and claim type and reviewer attribution (β=–0.074, *P*=.04) were all significant.

**Conclusions:**

Negative review features and the physician response significantly influence consumer choice through the causal attribution to physicians and reviewers. Physician attribution has a greater effect on consumers’ physician selection intention than reviewer attribution does. The presence of a physician response decreases the influence of negative reviews through direct and moderating effects. We propose some practical implications for physicians, health care providers, and online medical service platforms.

## Introduction

### Background

The user-generated content (UGC) mechanism provides patients an opportunity to post online reviews about physicians’ services [1]. These online physician reviews (OPRs) are attracting increasing consumer attention in recent years. In the beginning, health consumers mainly referred to these OPRs to book physicians offline [2]. With the development of online medical services, OPRs have become the most influential and important information source for selecting online physicians [3,4]. Particularly in the past few years, the COVID-19 pandemic has boosted the use of online medical services. Many consumers tend to seek online medical services to avoid physical touch, and they gradually get used to such services. Data from the Cyberspace Administration of China show that the number of online medical service users increased from 214 million in December 2020 to 300 million in June 2022, and 50% are new users with 1-2 years of experience [5]. In the consumerism era, the growing popularity of online medical services creates a need for physicians to embrace their online reputation and understand consumers’ decision processes, even though they have an unfavorable opinion of OPRs [6,7]. Therefore, an important question needs to be answered: How do OPRs affect consumers’ physician selection process in the online medical service context? The answer will be helpful for physicians to better manage their online reputation and promote their online performance.

Researchers have noted the OPR phenomenon and carried out studies on consumers’ evaluation of OPRs for physician selection [8,9]. A few studies have mainly focused on the review valence and confirmed the effectiveness of OPRs. McBride [10] found that parents are more likely to choose a neighbor-recommended physician with positive online ratings than a recommended physician with no rating information. Online review valence is positively related to consumer choice, and negative reviews have a greater influence than positive reviews [11,12]. Some other studies have investigated the effect of different numerical ratings on consumer choice. Han et al [13] reported that the rating and review quantity affect consumer choice through perceived physician trustworthiness. Lu and Wu [14,15] found that the number of reviews is more effective in influencing patient decisions compared to the overall rating and that disease risk moderates the relationship between the service quality score and patient choice. Li et al [4,16] also found that a high online rating has a positive and significant effect on patient utility. Chen and Lee [17] noted that a physician’s average rating increases the physician’s patient flow and that rating credibility moderates the positive effect. Additionally, textual review features have been investigated as they can improve the predictive power of patient choice [18,19]. A randomized experiment found that the association of review style with review quantity affects users’ attitude toward the rated physician [20]. The position, proportion, and length of negative physician reviews can also affect health consumers’ selection intention [21,22].

Although studies have provided insights into the understanding of health consumers’ decision-making behavior in the OPR context, there are still some important issues that need to be further explored. First, few studies have examined the effect of textual negative review features on helping narrow down consumer choice in the second stage of decision-making. As negative OPRs are inevitable for physicians, who worry that even 1 negative review can affect patients’ choice and damage their reputation [23], more negative OPR features need researchers’ attention. Second, the mechanism of how negative reviews work is overlooked. Although some studies have investigated the direct effect of textual review features [20,21], knowledge about how these reviews work is still limited. Investigating the mechanism will help us better understand the effect and provide more insights for stakeholders. Third, physician responses to OPRs are seldom discussed in consumers’ decision-making process. To manage negative reviews, physicians are actively involved in online services and provide necessary responses to save their reputation [24]. Whether and how the presence of a physician response plays a positive role in promoting their performance needs further investigation.

To address gaps in the literature, drawing from attribution theory, our study aimed to explore the underlying mechanism of how negative review features and the physician response affect consumers’ behavior in the online medical service environment. The main objectives of our study were threefold: Our first objective is to investigate the relationship between negative review features and consumers’ physician evaluation. The second objective is to validate the causal locus attribution that mediates the relationship between negative review features and physician selection behavior. The last objective is to assess whether the physician’s response moderates the effect of negative review features on a different locus attribution. Given that eHealth services are increasingly popular, we tested these arguments in the online medical service context to provide insights for stakeholders.

### Literature Review and Theoretical Background

#### Negative Online Review Features

An online review is a negative or a positive statement made by former consumers about a product, which is available to numerous people via the internet [25]. Negative information is considered more informative and diagnostic than positive information, and negative cues attract more attention than positive ones [26]. Consumers always place more emphasis on negative reviews when making a judgment or purchase decision [27,28]. When the decision-making process focuses on the textual message, positive framing is less effective than negative framing [29]. In this sense, negative reviews always have a stronger influence on consumers’ purchase intention than positive reviews do [11,30,31]. Thus, research always focuses on the influence of negative reviews on consumers’ decisions.

Specifically, prior studies have focused on several features of negative textual reviews, such as quantity [32], emotional intensity [33], severity of failure [34,35], proportion [25,34,36,37], quality [25,36], type [38], claim [39], and presentation order [32]. All these features can significantly affect consumer evaluation. In the online medical service context, physicians are no less worried about negative reviews than online merchants, even though most OPRs are positive [40]. Physicians worry that patients’ subjective reviews will damage their reputation, and the proportion and claims of negative OPRs are the main concern [7]. To decrease physicians’ concerns and provide insights into the understanding of consumers’ information processing progress, this study aimed to examine whether the proportion and claim type of negative OPRs influence consumer choice through different mechanisms, particularly under the condition in which the physician’s overall rating is high.

#### Physician Responses

As negative reviews have a notable impact on consumers’ attitude and final decision [41], companies are increasingly using responses to reviews as a service recovery method to respond and apologize to complainants [41]. Responses to negative reviews can be understood as a service carried out to cocreate value with consumers and other stakeholders [42]. Regardless of the response strategy, managerial responses can create external effects on negative review posters and potential consumers [43]. Responses to negative reviews also mitigate the adverse effects of the negative reviews on the sellers’ reputation and positively influence consumers’ trust toward the sellers, as well as consumers’ evaluation of the products and services [44].

Similar to online sellers, physicians are also under the threat of negative reviews. With increasingly more OPRs being generated, physicians have to respond to negative reviews to protect their reputation and decrease the negative effects. However, few studies have investigated the effect of physician responses. From a health consumer’s perspective, since they try to make use of further information about the rated physician from the responses, the responses serve as an important cue for evaluation and decision-making. If there is no physician response, consumers can only rely on negative reviews, which may increase their concerns. As both negative reviews and managerial responses can affect consumers’ evaluating process, there is a need to examine the effects of negative OPRs and physician responses, as well as the interactive effect on consumer behavior. Specifically, this study aimed to examine the direct and indirect effects of physician responses on consumers’ final choice.

#### Attribution Theory

Attribution theory is a social cognition theory that addresses causal explanations and reasoning about the expressions and actions of another person [45]. This theory provides an understanding of how individuals interpret incidents based on their causal inferences, and it has been widely used in management and marketing research to explain individuals’ evaluation processes [46,47]. In particular, attribution theory is an effective approach to understanding unfavorable incidents [48], such as negative employee reviews [49] and service failures [50]. Attribution theory has three dimensions, namely locus of control (internal or external), stability (stable or unstable), and controllability (controllable or uncontrollable) [48], and researchers often use different dimensions of the theory to fit their research questions and contexts [46]. In this study, we mainly focused on the “locus of control” dimension. The locus of causality establishes the cause as residing within the person (internal) or outside the person (external). The locus of control is related to who is to blame for undesirable or negative experiences [51].

In the OPR context, when health consumers encounter a negative OPR, they seek the underlying cause and show specific attitudes or emotions toward the physician, which affects their subsequent evaluation and selection behavior. In general, readers mainly attribute the origin of a negative review to the reviewer (external attribution) or the rated physician (internal attribution). However, how health consumers form different attribution loci is still unclear. Thus, for a deeper understanding of the psychological process that drives consumers’ online physician selection behavior, this study further introduced physician attribution and reviewer attribution as the mediating variable in the link between negative review features and consumer choice.

## Methods

### Research Model and Hypothesis Development

**Figure 1:**
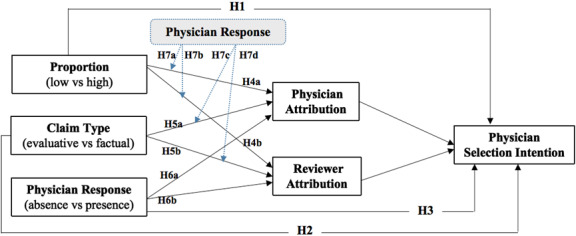
Proposed research model and hypotheses. H: hypothesis.

Based on the literature and attribution theory, we constructed our research model (Figure 1). We examined the effects of negative review features on consumers’ physician selection intention (hypothesis 1 [H1], H2). Furthermore, we investigated the mediating role of the attribution locus in the relationship between review features and consumer choice (H4, H5, H6) and how the presence of a physician response plays a role (H3, H7).

### Negative OPRs and Physician Selection Intention

Although the majority of OPRs are positive, it is inevitable for physicians to receive negative reviews. In the era of information overload, the existence of large volumes of positive reviews makes consumers pay more attention to limited negative reviews [27]. The negative review features provoke consumers’ deep evaluation in the second stage of decision-making after they have narrowed down their choices by rating and review quantity in the first stage.

The proportion of negative reviews is the number of negative reviews compared with overall online reviews. It is an important variable consumers consider when evaluating the online reviews of a desired object [52]. An increase in negative reviews escalates consumers’ perception of product risk and in turn depresses their purchase intention [25,53]. A previous study confirmed that consumers’ attitude lessens when the negative online review proportion rises [36]. In the OPR context, consumers’ physician selection intention decreases as the negative review proportion increases, even if the physician’s overall rating is relatively high. Based on these findings, we set out the first hypothesis (H1):

H1: A high proportion of negative OPRs decreases consumers’ physician selection intention more than a low proportion of reviews.

The other review feature that affects consumers’ judgment is the claim type of a review: evaluative or factual. An evaluative claim includes emotional, subjective impressions or opinions about product attributes, whereas a factual claim includes numeric values and objective facts about product attributes [54]. The definitions indicate that the 2 types of claims differ in the levels of objectivity and verifiability [55]. Factual claims are easily verifiable and provide detailed indicators of the performance [56], while evaluative claims are susceptible to multiple interpretations and are often considered not objective. Therefore, consumers are likely to perceive factual reviews as more helpful for their decision-making than evaluative claims [57]. In the OPR context, some patients write detailed factual descriptions about their negative medical experiences, while other patients just express their anger and negative emotions. For a physician with a high overall rating, factual claims in negative reviews are more persuasive than evaluative claims. Hence, we reasonably hypothesized the following:

H2: Factual negative OPRs decrease consumers’ physician selection intention more than evaluative reviews do.

Responses to negative reviews are usually written positively to reduce the impact of the reviews. In e-commerce settings, a seller’s apology and explanation of the cause of the service failure can be perceived as a repair effort by readers [58]. The responses provide additional and reasonable information for consumers’ evaluation process. Prior studies have shown that responses have positive effects on complainants’ repurchase intentions [59], and they can affect the extent to which consumers are satisfied with service recovery [60]. The responses of high-rated physicians who receive negative reviews demonstrate their responsibility and effectively decrease the influences of the negative reviews. However, an absence of responses increases information asymmetry and consumers’ risk perception. Therefore, we proposed the following hypothesis:

H3: The presence of a physician response increases consumers’ selection intention more than the absence of a physician response does.

### The Mediating Role of Physician Attribution and Reviewer Attribution

According to attribution theory, people always try to assess to what extent a certain entity is responsible for a negative or unexpected event [61]. Applied to the context of negative OPRs, health consumers always assess who is responsible for the negative reviews they encounter. The “locus of control” concept indicates that a negative review is caused mostly by either internal factors or external factors [61]. On the one hand, consumers may attribute a negative OPR to physician-related factors. In this case, consumers believe the negative information reflects what actually happens. On the other hand, consumers may attribute the cause or responsibility to reviewer-related factors, such as their motivations, moods, or bias [46,62]. In this case, the negative review is not (or is less) perceived as reflecting reality but rather as the reviewer’s personal subjective or prejudiced feeling. Due to information uncertainty and the complexity of medial issues, health consumers often attribute the causes of a negative review to both the physician and the reviewer, although to different degrees. When the level of physician attribution increases, consumers’ willingness to select the physician decreases. On the contrary, consumers’ physician selection intention increases when the level of reviewer attribution increases.

Regarding the formation of a different locus attribution, specific negative review features play an important role. In this study, the negative review proportion, claim type, and physician response may have an influence on consumers’ attribution judgment.

Consumers’ evaluation is based on their perceived conformity of reviews. A prior study on conformity research indicated that the greater the number of people with the same opinion, the greater the level of conformity to that opinion [36]. In other words, people are always influenced by the majority of the group, and the proportion of negative reviews from others could be a critical criterion for their attribution judgment. For a physician with a high overall rating, a higher proportion of negative reviews makes consumers believe the credibility of those reviews and confirms that the negative reviews are caused by the physician. Thus, consumers’ physician attribution level increases and reviewer attribution level decreases. In the case of a low proportion of negative reviews, consumers trust positive reviews posted by others more and tend to believe that negative reviews are caused by reviewer-related factors. Thus, consumers’ physician attribution level decreases and reviewer attribution level increases. As physician attribution and reviewer attribution are related to consumers’ physician selection intention, we decided to also examine the mediating effects of physician attribution and reviewer attribution in our study. Therefore, we proposed the following hypotheses:

H4a: Physician attribution mediates the effect of the negative review proportion on consumer choice (proportion +→ physician attribution –→ physician selection intention).

H4b: Reviewer attribution mediates the effect of the negative review proportion on consumer choice (proportion –→ reviewer attribution +→ physician selection intention).

Review contents show detailed reasons from reviewers, and different review claim types have different degrees of persuasiveness [20]. Factual negative reviews describe the objective facts or results related to a physician’s service, and these factual claims have high levels of credibility and verifiability. In this case, consumers tend to believe the negative claims to be true facts, and the physician needs to be responsible for the negative experience. Thus, consumers’ physician attribution level increases and reviewer attribution level decreases. On the contrary, evaluative reviews reflect reviewers’ subjective, emotional opinions and impressions about the physician [39]. The nonevidence-based reviews are considered an inaccurate and imprecise evaluation of the physician’s performance. As the physician’s overall rating is high, it is easy to attribute the negative reviews to the reviewers’ personal bias. Therefore, consumers’ physician attribution level decreases and reviewer attribution level increases. Considering the relationship between locus attribution and physician selection intention, we can reasonably believe that the attribution locus mediates the relationship between review claim type and consumer choice. Hence, the following hypotheses were proposed:

H5a: Physician attribution mediates the effect of the negative review claim type on consumer choice (claim type +→ physician attribution –→ physician selection intention).

H5b: Reviewer attribution mediates the effect of the negative review claim type on consumer choice (claim type –→ reviewer attribution +→ physician selection intention).

Most negative OPRs derive from misunderstandings between physicians and patients. The physician response can help develop a better relationship with complainants and create an external effect on potential consumers [43]. Both negative reviews and physician responses can provide insights into physician service. Physician responses provide additional information for potential consumers to make decisions, in addition to negative reviews. If there is no physician response, consumers can only use one-sided negative information to make causal attribution judgments, and the likelihood of physician attributing increases. A physician response not only shows the physician’s responsibility but also reduces misunderstanding. In this case, consumers’ physician attribution level is likely to decrease and their reviewer attribution level is likely to increase. Thus, a physician response makes consumers perceive a lower level of physician attribution than no response, while it has the opposite effect on reviewer attribution. As physician attribution and reviewer attribution are related to consumers’ selection intention, the 2 locus attributions can be said to mediate the effect of the physician response on consumer choice. Based on this, the study hypothesized the following:

H6a: Physician attribution mediates the effect of the physician response on consumer choice (physician response –→ physician attribution –→ physician selection intention).

>H6b: Reviewer attribution mediates the effect of the physician response on consumer choice (physician response +→ reviewer attribution +→ physician selection intention).

### The Moderating Role of Physician Responses

Responses to negative reviews can not only directly influence consumer attitude [49] but also indirectly decrease the effect of negative reviews on consumers’ intention. As a communication strategy, managerial responses are targeted at problem solving and trust remedy. Responses are expected to reduce the unfavorable impacts of negative reviews on both potential and existing consumers [63]. Some studies have confirmed the moderating effect of responses on the relationship between negative reviews and sellers’ performance. For example, responses have been found to moderate the effect of negative reviews on booking intention [64] and the number of bookings [65]. Different from prior studies, we aimed to explore the moderating effects of managerial responses (absence or presence) on the relationship between negative review features and locus attribution. We proposed the following hypotheses:

H7a/H7b: The impact of the proportion of negative reviews on physician attribution/reviewer attribution decreases/increases in the presence of a physician response compared to the absence of a physician response.

H7c/H7d: The impact of factual negative reviews on physician attribution/reviewer attribution decreases/increases in the presence of a physician response compared to the absence of a physician response.

### Study Design

Medical websites provide many physicians’ rating information for consumers, including ratings, review quantity, and textual reviews. Health consumers tend to narrow down their choice set by numerical rating information in the first stage, while they deliberately read the textual reviews to make the final decisions in the second stage [13]. To examine the effect of negative textual reviews, this study used a 2 (negative review proportion: low vs high) × 2 (negative review type: evaluative vs factual) × 2 (physician response: absence vs presence) between-subject design in a decision-controlled setting, where participants were asked to select a physician from a fictitious website designed for the purpose of the experiment. A pretest and a main experiment were conducted. The pretest was used to assess the appropriateness of the stimulus manipulation, and the main experiment was used to test the effects of stimulus features.

#### Stimulus Design

To provide the participants with a realistic context, we designed a physician selection scenario and the fictitious stimulus resembled a famous medical website in China (91160 [66]). The website has more than 659,000 physicians and has served 814 million person-times up to September 2023. The characteristics of this website can be generalized to other medical websites. The selected physician works in a high-level hospital in a city, and we used a physician icon that did not show any additional features of the physician in order to control the influence of the physician’s image. For textual reviews, we presented the newest 10 of 35 reviews on the first page. The detailed fictitious reviews are shown in Multimedia Appendix 1.

##### Proportion of Negative Reviews

Prior studies have considered 8 online reviews in their research [25,36]. Most experiments have used 12.5% or 25% as the low-proportion threshold and 50% or 62.5% as the high-proportion threshold [25,36,37]. One study in the OPR context used 1/6, 2/6, 3/6, 4/6, 5/6, and 6/6 to represent different levels of the negative review proportion [21]. In our study, participants were presented with 10 textual reviews. This design is prevalent on Chinese websites. When designating the proportion of negative reviews, we not only referred to prior research [25,36,37] but also considered the realistic situation of physician-rating websites. In a simulated page containing 10 textual reviews, 1 negative review is the minimal unit, which is common in realistic situations. Thus, we set 10% as a low proportion, which is also closer to prior research. Considering that physicians always get a few negative reviews on real physician-rating websites, we did not use a proportion exceeding 50% to represent a high proportion as in previous studies. Instead, after investigating a large number of physician reviews on the 91160 website [66], we think that designating a ratio of over 50% as a high proportion is not in line with the actual situation. Therefore, we chose 3 of 10 (30%) to represent a high proportion. It is not only different from the ratio of 10% but also conforms to reality. This designation is also more realistic than previous experiments. To control the influence of the negative review position [21], we set the negative reviews at 3rd, 6th, and 8th place in the high-proportion group and the 5th place in the low-proportion group.

##### Claim Type of Negative Reviews

All the 10 reviews in the manipulated setting were real reviews posted on the 91160 website [66], which increased the external validity. With the exception of the claim type and review valence, the reviews were identical, containing evaluations about physicians’ skills, effect, and attitude. For a positive review, all attributes were described positively, whereas for a negative review, all attributes were described negatively. Additionally, factual claims included detailed descriptions of patients’ experiences and the effects, whereas evaluative claims focused on patients’ subjective feelings about attributes (unfriendliness, ineffective, incompetence).

##### Physician Responses

In this study, we only examined how the presence or absence of physician responses to negative reviews affects consumer evaluation. In the response absence scenario, the physician provides no response to any negative or positive review. In the response presence scenario, the physician provides a necessary explanation and encourages the reviewer to visit for further treatment again. This response strategy is widely used by physicians on the 91160 website [66]. Finally, 8 versions of web pages containing numerical rating information and textual reviews were created.

#### Pretest

We also conducted a pretest with 72 participants to assess the appropriateness of the stimulus manipulation. These participants were convenience samples recruited via researchers’ WeChat, and they all had used OPRs. The participants were asked to rate a physician proposal for a neighbor, and each participant was randomly assigned to 1 alternative proposal. They used a 7-point Likert scale to rate the physician on 2 aspects: resemblance of the physician proposal to the 91160 website and understandability of the textual review information.

#### Main Experiment

First, we explained the purpose of the experiment. Next, during the experiment, we presented the following brief scenario:

Aunt Wang is your familiar neighbor, and she has a stomachache for a few days. She finds a physician on the 91160 website, which is a widely used medical website. You are requested to help her decide whether the physician is the right choice.

As Chinese people often help friends and family members select physicians, the scenario is prevalent in the real world. Next, we presented the physician’s picture, which included only personal information and a numerical rating. The physician’s rating was 9.1, and the review quantity is 35, which are average values on the 91160 website [66]. Participants were asked to report their intention to select the physician before they were exposed to negative textual reviews. Finally, 10 textual reviews of the physician were added. Participants were requested to read the reviews carefully and then complete questions about the manipulation effect, physician attribution, reviewer attribution, and physician selection intention. This procedure was consistent with the steps health consumers take to select a physician in the online environment.

### Participants

We used the snowballing method to recruit participants. As students are not the main online medical service users, they were excluded in the first-round survey. First, 50 participants with different genders, ages, education levels, living cities, and career backgrounds were recruited via researchers’ WeChat. Second, the 50 participants were requested to invite their WeChat friends with different socioeconomic statuses to finish our online experiment. We also used survey quotas based on gender, age, and education level, ensuring a higher diversity of the sample compared to standard convenience sampling techniques. All sessions of the experiment were conducted online, and participants were randomly assigned to 1 of 8 experimental scenarios.

### Ethical Considerations

The study was approved by the Ethics Committee of the Guangdong University of Finance & Economics. All participants provided informed consent upon registration for the study. Participants were ensured that their privacy would be protected and their answers would only be used for academic research. Each participant was paid RMB 3 (US $0.42) after they conscientiously completed the questionnaire.

### Measures

The dependent variable was physician selection intention, which was assessed using 3 items rated on a 7-point scale (ranging from 1 for completely disagree to 7 for completely agree). The scale was adapted from previous studies [13,21]. Physician attribution and reviewer attribution were also measured and included as mediating variables. The 2 constructs were adapted from a prior study [34]. For subsequent data analyses, we calculated the mean scores for all items of each construct.

After the questionnaire was initially developed, we invited 3 experts to assess content accuracy, validity, and ease of understanding before the experiment began. Additionally, a pilot test was conducted with 15 participants to verify the clarity of the questions and 2 items were slightly modified based on their feedback.

### Manipulation Check

Based on previous studies [67,68], we conducted a manipulation check. Every participant was asked to rate the proportion of negative reviews, the negative review claim type, and the physician response.

### Data Analysis

Using IBM SPSS Statistics and SmartPLS3, we applied 1-way ANOVA and partial least squares–structural equation modeling (PLS-SEM) to test the proposed hypotheses. PLS allows the simultaneous testing of mediation with minimum bias, resulting in greater appreciation of the complete effect. The method is better than simple linear regression, in which the mediation pathway is tested individually [69]. We conducted bootstrapping with 5000 resamples to obtain the *t* statistics and SEs. We also performed a full collinearity test to determine whether any constructs contained variance inflation factor (VIF) values of ≥3.3.

## Results

### Participant Details

In total, 550 participants provided complete data, and 91 (16.5%) surveys were discarded for too short response times or same values of all continuous measurement scales. Finally, 459 (83.5%) valid questionnaires were collected, with samples ranging from 51 to 67 (11.1%-14.6%) in the 8 experimental scenarios (Table 1). These participants were randomly grouped according to the last digit of their ID card number. The chi-square test verified that there were no significant differences (*P*=.24) in the characteristics of the participants among the 8 experimental scenarios.

**Table 1:** Details of the 8 experimental scenarios, including participants (N=459).

Scenario	Proportion of negative reviews (N=10), n (%)	Claim type	Response	Sample, n (%)
1	1 (10)	Evaluative	Absent	67 (14.6)
2	3 (30)	Evaluative	Absent	51 (11.1)
3	1 (10)	Factual	Absent	53 (11.5)
4	3 (30)	Factual	Absent	57 (12.4)
5	1 (10)	Evaluative	Present	55 (12.0)
6	3 (30)	Evaluative	Present	56 (12.2)
7	1 (10)	Factual	Present	59 (12.9)
8	3 (30)	Factual	Present	61 (13.3)

### Demographic Characteristics

Participants’ demographic characteristics are shown in Table 2. Of the 459 participants, 280 (61%) were 31-45 years old, 226 (49.2%) were female, and 262 (57.1%) were married. In addition, 314 (68.4%) participants had completed a college or higher level of education, and 323 (70.4%) had a monthly income of RMB 3001-12,000 (US $416.89-$1667.01).

**Table 2:** Demographic characteristics of the sample (N=459).

Characteristics		Participants, n (%)
**Age (years)**		
	23-30	129 (28.1)
	31-35	156 (33.9)
	36-45	124 (27.1)
	≥46	50 (10.9)
**Gender**		
	Male	233 (50.8)
	Female	226 (49.2)
**Daily internet usage (hours)**		
	≤3	15 (3.3)
	3 to <7	260 (56.6)
	7 to <11	78 (17.0)
	11 to ≤13	106 (23.1)
**Monthly income (RMB)^a^**		
	≤3000 (US $416.75)	96 (20.9)
	3001-8000 (US $416.89-$1111.34)	172 (37.5)
	8001-12,000 (US $1111.48-$1667.01)	151 (32.9)
	≥12,001 (US $1667.15)	40 (8.7)
**Highest education**		
	Middle school	145 (31.6)
	College/bachelor	154 (33.6)
	Master/PhD	160 (34.8)
**Marital status**		
	Married	262 (57.1)
	Single	197 (42.9)

^a^An exchange rate of RMB 1.00=US $0.14 has been used.

### Pretest

In the pretest to assess the appropriateness of stimulus manipulation, the mean score of resemblance was 6.47 (SD 0.94), and the mean score of review understandability was 5.78 (SD 1.07). No significant differences (*P*=.32) were observed between 3 manipulated factor designs in resemblance and understandability.

### Manipulation Check

ANOVA results indicated that participants in the high-proportion group rated the proportion of negative reviews as higher compared to the low-proportion group (mean_1_ 5.700, SD 0.947; mean_2_ 2.290, SD 0.960; *F*_1,457_=1464.039, *P*<.001). Participants exposed to factual negative reviews perceived the reviews as more objective than participants exposed to evaluative negative reviews (mean_1_ 5.440, SD 1.030; mean_2_ 2.640, SD 0.934; *F*_1,457_=930.495, *P*<.001). Participants exposed to response presence scenarios perceived a higher level of response than those exposed to a response absence scenario (mean_1_ 5.540, SD 1.052; mean_2_ 2.530, SD 0.918; *F*_1,457_=1065.037, *P*<.001). No other effects were significant. Thus, the manipulation was successful.

### Model Assessment

We used internal consistency reliability, convergent validity, and discriminant validity to examine measures. As Table 3 shows, composite reliability (CR) values (above the lower limit of 0.7) indicated internal consistency reliability. As standardized factor loadings were >0.7 at a significance level of .001 and the average variance extracted (AVE) values were well above 0.5 [70], the convergent validity was supported. Furthermore, as the heterotrait-monotrait (HTMT) ratio of the correlations was lower than the threshold of 0.85 and the HTMT CIs did not include 1.0, all constructs exhibited discriminant validity. Thus, the results provide supportive evidence for the constructs’ reliability and validity.

**Table 3:** Assessment of reliability and convergent validity.

Construct and items			Mean (SD)		Loading		AVE^a^	CR^b^		Cronbach α
**Physician attribution (PA)**							0.772	0.910		0.852
	PA1	3.08 (1.524)		0.882		—^c^		—	—	
	PA2	3.20 (1.507)		0.888		—		—	—	
	PA3	3.09 (1.522)		0.866		—		—	—	
**Reviewer attribution (RA)**							0.753	0.901		0.836
	RA1	4.78 (1.440)		0.865		—		—	—	
	RA2	4.62 (1.494)		0.885		—		—	—	
	RA3	4.38 (1.461)		0.853		—		—	—	
**Physician selection intention (PSI)**							0.903	0.945		0.917
	PSI1	4.42 (1.772)		0.920		—		—	—	
	PSI2	4.41 (1.808)		0.927		—		—	—	
	PSI3	4.43 (1.750)		0.930		—		—	—	

^a^AVE: average variance extracted.

^b^CR: composite reliability.

^c^Not applicable.

Additionally, the standardized root mean square residual value for the structural model was <0.08 (0.076 for our model), which indicated good model fit. The *R*^2^ values for physician attribution (*R*^2^=0.221), reviewer attribution (*R*^2^=0.416), and consumers’ physician selection intention (*R*^2^=0.517) were all larger than 0, supporting the predictive relevance of the model.

### Collinearity Test

Data analysis results showed that pathological VIFs for all constructs ranged from 1.586 to 2.036, confirming that common method biases are not a threat for our study.

### Hypotheses Test

#### Main Effects

Before the hypotheses test, we used paired-sample *t* tests to compare consumers’ physician selection intention before and after they were exposed to negative textual reviews (Table 4). According to the *t* test results, consumers reported lower levels of physician selection intention after reading the detailed negative reviews (mean_before_ 5.059, SD 1.493; mean_after_ 4.419, SD 1.645; *P*<.001).

**Table 4:** Results of the t test of the main effects on the physician selection intention.

Review features		Mean (SD)	*P* value
**Negative review exposure**			<.001
	Before	5.059 (1.493)	—^a^
	After	4.419 (1.645)	—
**Review proportion**			<.001
	High	3.760 (1.715)	—
	Low	5.053 (1.292)	—
**Claim type**			<.001
	Factual	3.805 (1.660)	—
	Evaluative	5.020 (1.391)	—
**Physician response**			<.001
	Present	4.774 (1.583)	—
	Absent	4.072 (1.645)	—

^a^Not applicable.

We conducted ANOVA to test the relationship between review features and physician selection intention (Table 4). The proportion of negative online reviews significantly influenced consumer choice (*F*_1,457_=83.618, *P*<.001). Participants in the high-proportion group (mean 3.760, SD 1.715) reported a significantly lower level of intention than those in the low-proportion group (mean 5.053, SD 1.292). Therefore, H1 was accepted.

The study yielded the same results for the effects of the claim type of negative reviews (Table 4). The claim type of negative reviews significantly affected consumer choice (*F*_1,457_=72.404, *P*<.001). Factual negative reviews (mean 3.805, SD 1.660) led participants to report a significantly lower level of selection intention than evaluative negative reviews (mean 5.020, SD 1.391). Thus, H2 was supported.

In addition, physician response also had a significant influence on consumers’ intention to select a physician with negative reviews (*F*_1,457_=21.849, *P*<.001), as shown in Table 4. Participants exposed to negative reviews with a physician response present (mean 4.774, SD 1.583) reported a significantly higher level of intention compared to the response absence scenario (mean 4.072, SD 1.645). Therefore, H3 was supported.

### Mediating Effects

**Figure 2:**
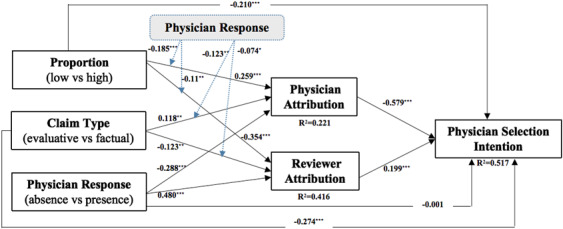
Mediation and moderation effects of the structural model.

Following the standard procedure applied in a previous study [69], we used the latent variable score generated in PLS-SEM as input for mediation and moderation effects. The results are shown in Figure 2.

Table 5 shows the significant direct and indirect effects of review features. As shown in Figure 2 and Table 5, physician attribution (β=–0.579, 95% CI –0.640 to –0.511, *P*<.001) and reviewer attribution (β=0.199, 95% CI 0.137-0.263, *P*<.001) induced by negative review features were both significantly related to physician selection intention. Their effects were opposite, and physician attribution had a greater influence than reviewer attribution. In addition, 3 review features also had significant effects on consumers’ locus attribution.

**Table 5:** Direct and indirect effects of review features on consumer choice.^a^

Hypothesis and parameters		Effect, β (95% CI)	SE	*t*_4.454_ Test	*P* value
**H^b^4a**					
	Proportion→PA^c^	0.259 (0.176 to 0.340)	0.041	6.253	<.001
	PA→PSI^d^	–0.579 (–0.640 to –0.511)	0.033	17.505	<.001
	Proportion→PA→PSI	–0.150 (–0.202 to –0.099)	0.026	5.701	<.001
**H4b**					
	Proportion→RA^e^	–0.354 (–0.419 to –0.286)	0.034	10.301	<.001
	RA→PSI	0.199 (0.137 to 0.263)	0.032	6.222	<.001
	Proportion→RA→PSI	–0.071 (–0.100 to –0.045)	0.014	5.079	<.001
**H5a**					
	Claim type→PA	0.118 (0.036 to 0.199)	0.042	2.778	.005
	PA→PSI	–0.579 (–0.640 to –0.511)	0.033	17.505	<.001
	Claim type→ PA→PSI	–0.068 (–0.118 to –0.021)	0.025	2.714	.01
**H5b**					
	Claim type→RA	–0.123 (–0.198 to –0.051)	0.037	3.330	.001
	RA→PSI	0.199 (0.137 to 0.263)	0.032	6.222	<.001
	Claim type→RA→PSI	–0.025 (– 0.045 to –0.009)	0.009	2.587	.01
**H6a**					
	Physician response→PA	–0.288 (–0.364 to –0.209)	0.040	7.280	<.001
	PA→PSI	–0.579 (–0.640 to –0.511)	0.033	17.505	<.001
	Physician response→PA→PSI	0.167 (0.118 to 0.214)	0.025	6.711	<.001
**H6b**					
	Physician response→RA	0.480 (0.417 to 0.542)	0.032	14.894	<.001
	RA→PSI	0.199 (0.137 to 0.263)	0.032	6.222	<.001
	Physician response→RA→PSI	0.096 (0.065 to 0.130)	0.016	5.815	<.001

^a^Results are based on bootstrapping with 5000 subsamples (2-tailed).

^b^H: hypothesis.

^c^PA: physician attribution.

^d^PSI: physician selection intention.

^e^RA: reviewer attribution.

First, the proportion of negative reviews was significantly and positively associated with physician attribution (β=0.260, *P*<.001) and significantly and negatively associated with reviewer attribution (β=–0.355, *P*<.001). The mediation tests also indicated that mediations are in play, whereby the proportion of negative reviews influenced physician attribution and reviewer attribution, which in turn affected consumer selection intention. The total indirect effect size of the proportion of negative reviews on consumers’ physician selection intention was –0.221 (95% CI –0.277 to –0.165, *P*=.02). Thus, H4a and H4b were supported.

Second, the negative review claim type was also significantly associated with physician attribution (β=0.118, *P*=.005) and reviewer attribution (β=–0.123, *P*=.001). Factual reviews induced a higher level of physician attribution and a lower level of reviewer attribution, while evaluative reviews induced a lower level of physician attribution and a higher level of reviewer attribution. Mediation analyses indicated that physician attribution and reviewer attribution significantly mediate the effects of the claim type on consumers’ physician selection intention. The total indirect effect size of the negative review claim type on consumers’ physician selection intention was –0.093 (95% CI –0.143 to –0.044, *P*=.02). Hence, H5a and H5b were supported.

Third, the physician response had a significantly opposite influence on physician attribution (β=–0.288, *P*<.001) and reviewer attribution (β=0.480, *P*<.001). Our mediation tests also indicated that physician attribution and reviewer attribution mediate the influence of the physician response on consumers’ physician selection intention. The total indirect effect size of the physician response on consumer choice was 0.262 (95% CI 0.206-0.317, *P*<.001). Therefore, H6a and H6b were supported.

Considering the total effect size of 3 review features, we found that the negative review proportion (β=–0.371, 95% CI –0.447 to –0.295, *P*<.001) and review claim type (β=–0.343, 95% CI –0.420 to –0.267, *P*<.001) have greater effects than the physician response (β=0.194, 95% CI 0.110-0.259, *P*<.001).

### Moderating Effects

The moderating effects of the physician response on the relationship between the negative review proportion and physician attribution (β=–0.185, *P*<.001), negative review proportion and reviewer attribution (β=–0.110, *P*<.001), claim type and physician attribution (β=0.123, *P*=.003), and claim type and reviewer attribution (β=0.074, *P*=.04) were all significant. The *R*^2^ change in physician attribution (0.053) and reviewer attribution (0.035) significantly increased after the moderator was added. The results also indicated that the physician response decreases the effects of negative review features on physician attribution but increases the effects of negative review features on reviewer attribution. The significant moderating effects permitted subsequent investigation of indirect effects. Bootstrapping with 5000 resamples revealed that the physician response plays a significant moderating role in all the indirect relationships of negative review features with physician selection intention (Table 6), with 95% CIs never straddling 0. Thus, H7a-H7d were all supported.

**Table 6:** Moderating effects of the physician response.

Hypothesis and parameters		Effect, β (95% CI)	SE	*t*_4.454_ Test	*P* value
**H^a^7a**					
	Physician response×proportion→PA^b^	–0.185 (–0.266 to –0.105)	0.041	4.499	<.001
	Physician response×proportion→PA→PSI^c^	0.107 (0.060 to 0.154)	0.024	4.468	<.001
**H7b**					
	Physician response×proportion→RA^d^	–0.110 (–0.183 to –0.040)	0.036	3.022	.003
	Physician response×proportion→RA→PSI	–0.022 (–0040 to –0.007)	0.008	2.610	.01
**H7c**					
	Physician response×claim→PA	–0.123 (–0.205 to –0.041)	0.042	2.937	.003
	Physician response×claim→PA→PSI	0.071 (0.022 to 0.118)	0.024	2.933	.003
**H7d**					
	Physician response×claim→RA	–0.074 (–0.147 to –0.002)	0.036	2.053	.04
	Physician response×claim→RA→PSI	–0.068 (–0.030 to –0.001)	0.026	2.463	.01

^a^H: hypothesis.

^b^PA: physician attribution.

^c^PSI: physician selection intention.

^d^RA: reviewer attribution.

## Discussion

### Principle Findings and Theoretical Contributions

The increasing proliferation of OPRs has enabled the wide usage of online medical services. Physicians should not only worry about negative reviews but also more actively obtain knowledge to better manage their online reputation [13,71]. To examine the effect of negative reviews, this study adopted attribution theory to analyze how review features influence consumers’ physician selection intention. Past studies have confirmed that online reviews can influence consumer choice through attribution evaluation [72-74]. This study differs from these prior studies as we focused on negative physician reviews and different locus attributions. It explains how 2 simultaneously stimulated locus attributions can mediate the relationship between review features and consumer choice in the eHealth context. This is an important addition to the previous literature on attribution studies because it offers a deep understanding of health consumers’ responses to negative reviews. The findings of this study provide insights into ways that physicians can best manage their negative online reviews. Several other key findings are worth noting.

First, the study shows that the proportion (high vs low) of negative OPRs influences consumer responses differently, and the influence is mediated by 2 coexisting locus attributions (physician attribution and reviewer attribution). A high proportion of negative OPRs significantly depresses consumers’ physician selection intention compared to a low proportion of negative OPRs. This finding confirms the results of prior studies [21,34,53,75], which state that consumers’ willingness tends to become more unfavorable as the proportion of negative reviews increases. However, the outcome is somewhat at odds with previous arguments that consumers’ purchase intention is indifferent to exposure to high or low proportions of negative online reviews of popular products [25]. This study extends the understanding by introducing 2 mediators that explain the mechanism of how consumers evaluate the negative review proportion. As people are always influenced by the majority of the group, high exposure to negative OPRs makes consumers believe that the reviews are true. Hence, negative reviews are more due to physician-related factors and less due to reviewer-related factors. These 2 different and coexisting causal locus attributions finally oppositely influence consumer choice.

Second, the claim type of negative OPRs also has an impact on consumers’ physician selection intention via different casual locus attributions. A prior study has demonstrated that writing styles used in online reviews determine both consumers’ perception of those messages and their choices [76], and researchers have obtained contradictory findings about the influence of review style on review acceptance [77]. In the OPR context, the review type also has an impact on how individuals evaluate the credibility of the review and the rated physician [78]. A previous study that suggested the interaction of review style and review quantity has an impact on consumers’ decision to accept a physician, while the direct effect of a positive review style is not significant [20]. The finding differs somewhat from our study, which indicates that a negative review style influences consumer choice through different causal attributions. Focusing on negative OPRs, our study extends previous research by identifying 2 new mediators (physician attribution and reviewer attribution), in addition to review credibility and helpfulness. The 2 mediators can better explain consumer choice when they encounter a high-rated physician with negative reviews with different styles.

Third, the physician response not only has a direct influence on consumers’ causal attribution evaluation but also moderates the effect of other negative review features.

As observers tend to make a default attribution to actors in a negative context, an appropriate response can provide additional information that leads consumers to acknowledge other possible causes and adjust default attribution [79]. In the OPR context, physicians with negative reviews often respond to help shift observers’ attribution of negative reviews away from them. Physician responses remind consumers that the reviews are not objective and the reviewers may be responsible for the negative reviews. Therefore, physician responses increase consumers’ attribution to the reviewers but decrease the attribution to physicians. This finding is similar to prior studies, which have confirmed that the effect is mediated by the reduced causal attribution of negative reviews to the firm [80]. However, our result is contrary to another study, which indicates that the presence of a response to negative reviews hurts consumers’ purchase intention [81]. In addition, the presence of a physician response also increases the effect of negative review features on reviewer attribution and decreases the effect of negative review features on physician attribution. Our result is in accordance with a previous study that showed that responses moderate the effect of negative reviews on consumers’ intention [64,65]. Our study also extends the findings of previous studies by simultaneously examining the direct and indirect effects of response presence and introducing a new path on which response presence plays a moderating role.

Finally, our study confirms that the effect of negative review features for a high-rated physician relies on how observers locate the cause of the negative reviews. The result can alleviate physicians’ worries that even 1 negative review can affect patient choice and damage physicians’ reputation [23]. For a physician with a relatively high rating, consumers further focus on negative reviews and make judgments about the cause after they make an initial decision based on the numerical rating information. This study examined the influence of numerical ratings and textual reviews in the same experiment and deepened our understanding of the consumer decision-making process.

### Practical Implications

An increasing number of physicians are providing online medical services as a way to extend their offline services. Understanding the influencing mechanism of negative reviews can help them embrace online services more confidently. Our results offer managerial implications for physicians, health care providers, and online medical service platforms.

First, physicians and health care providers need to obtain more textual reviews as a way to reduce the proportion of negative reviews. Review quantity is positively related to review ratings [2], and more reviews can decrease the visibility of negative reviews. A low proportion of negative reviews increases consumers’ causal attribution to reviewers and increases consumers’ positive attitude. Second, physicians and health care providers should actively and appropriately respond to negative reviews. The presence of responses to negative reviews plays an important role in consumers’ attribution allocation. Managerial responses not only greatly decrease physician attribution but also reduce the negative effects of reviews on physicians. There is no need for physicians to worry about negative reviews, if they provide effective responses that can activate consumers’ causal attribution to the reviewers. Reviewer attribution helps improve the patient-physician relationship. Third, online medical service platforms should display negative OPRs appropriately and remind physicians to strengthen communication with patients and manage their negative reviews. Negative online reviews are important information sources for consumers’ decision-making, and their position can greatly affect physicians’ reputation. Online medical platforms should also enable consumers to notice negative OPRs among numerous reviews, while avoiding having unfair impacts on physicians’ reputation.

### Limitations

Even through the study was conducted with methodological rigor, there are still several limitations that deserve to be included in future research. First, the physician in our scenario is a high-rated physician. Although a high-rated physician is closer to reality [2], this may raise concerns in terms of the generalization of results to a low-rated physician. Future research may explore the hypotheses in the context of a low-rated physician. Second, we only tested the effects of the proportion and claim type of negative reviews for a physician. Other features, such as severity, length, position, and criticism target of negative reviews, were overlooked and deserve further investigation. Third, the response strategy in this study provides necessary explanations and encourages the reviewer to visit for further treatment again. Even though this strategy is prevalent on physician-rating websites, there are other different response styles that may affect the effectiveness of responses [82]; this also holds potential for future investigation. Fourth, sample bias may affect the generalizability of research results. The convenient samples for this study mainly came from relatively young Chinese urban residents, who have a rich experience of using online reviews, and it is unclear whether the results are applicable to rural residents, who seldom use online reviews. Future research needs to analyze the evaluation behavior of this population.

### Conclusion

The goal of this research was to understand how negative review features and physician responses affect consumer choice. The results show that the proportion of negative reviews (high vs low), the claim type of negative reviews (factual vs evaluative), and the physician response (presence vs absence) can affect consumers’ physician selection intention through 2 different causal attributions (physician attribution and reviewer attribution). Physician attribution has greater influence on consumer choice than reviewer attribution does. The presence of a physician response also moderates the relationship between negative review features and causal attribution. We also provide some practical implications for physicians to manage their online reputation.

## Appendix

Multimedia Appendix 1 Questionnaire.

## Abbreviations

AVE: average variance extracted
CR: composite reliability
HTMT: heterotrait-monotrait
OPR: online physician review
PLS-SEM: partial least squares–structural equation modeling
VIF: variance inflation factor

## Data Availability

Data will be made available on request.

## References

[1] Liu JJ, Matelski J, Cram P, Urbach DR, Bell CM (2016). Association between online physician ratings and cardiac surgery mortality. Circ Cardiovasc Qual Outcomes.

[2] Han X, Li B, Zhang T, Qu J (2020). Factors associated with the actual behavior and intention of rating physicians on physician rating websites: cross-sectional study. J Med Internet Res.

[3] Hanauer DA, Zheng K, Singer DC, Gebremariam A, Davis MM (2014). Public awareness, perception, and use of online physician rating sites. JAMA.

[4] Li X, Chou S, Deily ME, Qian M (2021). Comparing the impact of online ratings and report cards on patient choice of cardiac surgeon: large observational study. J Med Internet Res.

[5] (2022). The 47th statistical report on internet development in China. Cyberspace Administration of China.

[6] Menon AV (2017). Do online reviews diminish physician authority? The case of cosmetic surgery in the U.S. Soc Sci Med.

[7] Samora JB, Lifchez SD, Blazar PE, American Society for Surgery of the Hand Ethics and Professionalism Committee (2016). Physician-rating web sites: ethical implications. J Hand Surg Am.

[8] Hanauer DA, Zheng K, Singer DC, Gebremariam A, Davis MM (2014). Parental awareness and use of online physician rating sites. Pediatrics.

[9] Ghosh S, Chakraborty S, Gupta N, Basu S (2023). What ails physician review websites? A study of information needs of patients. Decis Support Syst.

[10] McBride DL (2015). Parental use of online physician rating sites. J Pediatr Nurs.

[11] Han X, Qu J, Zhang T (2019). Exploring the impact of review valence, disease risk, and trust on patient choice based on online physician reviews. Telemat Informat.

[12] Carbonell G, Meshi D, Brand M (2019). The use of recommendations on physician rating websites: the number of raters makes the difference when adjusting decisions. Health Commun.

[13] Han X, Du JT, Zhang T, Han W, Zhu Q (2021). How online ratings and trust influence health consumers’ physician selection intentions: an experimental study. Telemat Informat.

[14] Lu W, Wu H (2019). How online reviews and services affect physician outpatient visits: content analysis of evidence from two online health care communities. JMIR Med Inform.

[15] Lu N, Wu H (2016). Exploring the impact of word-of-mouth about physicians' service quality on patient choice based on online health communities. BMC Med Inform Decis Mak.

[16] Li Y, Ma X, Song J, Yang Y, Ju X (2019). Exploring the effects of online rating and the activeness of physicians on the number of patients in an online health community. Telemed J E Health.

[17] Chen Y, Lee S (2023). User-generated physician ratings and their effects on patients’ physician choices: evidence from Yelp. J Mark.

[18] Xu Y, Armony M, Ghose A (2021). The interplay between online reviews and physician demand: an empirical investigation. Manag Sci.

[19] Wan Y, Peng Z, Wang Y, Zhang Y, Gao J, Ma B (2021). Influencing factors and mechanism of doctor consultation volume on online medical consultation platforms based on physician review analysis. Internet Res.

[20] Grabner-Kräuter S, Waiguny MKJ (2015). Insights into the impact of online physician reviews on patients' decision making: randomized experiment. J Med Internet Res.

[21] Li S, Feng B, Chen M, Bell RA (2015). Physician review websites: effects of the proportion and position of negative reviews on readers' willingness to choose the doctor. J Health Commun.

[22] Hu Y, Zhou H, Chen Y, Yao J, Su J (2021). The influence of patient-generated reviews and doctor-patient relationship on online consultations in China. Electron Commer Res.

[23] Schulz PJ, Rothenfluh F (2020). Influence of health literacy on effects of patient rating websites: survey study using a hypothetical situation and fictitious doctors. J Med Internet Res.

[24] Liu X, Hu M, Xiao BS, Shao J (2022). Is my doctor around me? Investigating the impact of doctors’ presence on patients’ review behaviors on an online health platform. J Assoc Inf Sci Technol.

[25] Shihab MR, Putri AP (2018). Negative online reviews of popular products: understanding the effects of review proportion and quality on consumers’ attitude and intention to buy. Electron Commer Res.

[26] Sperlich PW (2014). Attribution: perceiving the causes of behavior. Edited by Edward E. Jones, David E. Kanouse, Harold H. Kelley, Richard E. Nisbett, Stuart Valins, and Bernard Weiner. (Morristown, N.J.: General Learning Press, 1972. Pp. 186. $8.95.). Am Polit Sci Rev.

[27] Skowronski JJ, Carlston DE (1987). Social judgment and social memory: the role of cue diagnosticity in negativity, positivity, and extremity biases. J Pers Soc Psychol.

[28] Sen S, Lerman D (2007). Why are you telling me this? An examination into negative consumer reviews on the Web. J Interact Mark.

[29] Maheswaran D, Meyers-Levy J (1990). The influence of message framing and issue involvement. J Mark Res.

[30] Nejad MG, Amini M, Sherrell DL (2016). The profit impact of revenue heterogeneity and assortativity in the presence of negative word-of-mouth. Int J Res Mark.

[31] Charlett D, Garland R, Marr N (1995). How damaging is negative word of mouth?. Mark Bull.

[32] Liao H, Huang Z, Liu S (2021). The effects of negative online reviews on consumer perception, attitude and purchase intention: experimental investigation of the amount, quality, and presentation order of eWOM. ACM Trans Asian Low-Resour Lang Inf Process.

[33] Wu Z, Guo T (2019). The effect of negative online review’s textual features on consumer’s purchase intention: analysis on the perspective of self-construal. Sci Technol Dev.

[34] Du X, Wu Y, Gao H, Li M (2021). Effect of negative online review and corporate response on customer purchase intention. J Syst Manag.

[35] S S, M.R A, Ponnam A (2018). Can online service recovery interventions benignly alter customers’ negative review evaluations? Evidence from the hotel industry. J Hosp Mark Manag.

[36] Lee J, Park D, Han I (2008). The effect of negative online consumer reviews on product attitude: an information processing view. Electron Commer Res Appl.

[37] Weisstein FL, Song L, Andersen P, Zhu Y (2017). Examining impacts of negative reviews and purchase goals on consumer purchase decision. J Retail Consum Serv.

[38] Joo B, Hwang S (2016). Impact of negative review type, brand reputation, and opportunity scarcity perception on preferences of fashion products in social commerce. Fash Bus.

[39] Kim M, Lee M (2015). Effects of review characteristics and consumer regulatory focus on perceived review usefulness. Soc Behav Pers Int J.

[40] Okike K, Peter-Bibb TK, Xie KC, Okike ON (2016). Association between physician online rating and quality of care. J Med Internet Res.

[41] Esmark Jones CL, Stevens JL, Breazeale M, Spaid BI (2018). Tell it like it is: the effects of differing responses to negative online reviews. Psychol Mark.

[42] Park S, Allen JP (2012). Responding to online reviews. Cornell Hosp Q.

[43] Wang Y, Chaudhry A (2018). When and how managers’ responses to online reviews affect subsequent reviews. J Mark Res.

[44] Ravichandran T, Deng C (2023). Effects of managerial response to negative reviews on future review valence and complaints. Inf Syst Res.

[45] Martinko MJ, Harvey P, Dasborough MT (2010). Attribution theory in the organizational sciences: a case of unrealized potential. J Organ Behav.

[46] Kelley HH, Michela JL (1980). Attribution theory and research. Annu Rev Psychol.

[47] Abascal TE, Fluker M, Jiang M (2016). Domestic demand for Indigenous tourism in Australia: understanding intention to participate. J Sustain Tour.

[48] Hastie R (1984). Causes and effects of causal attribution. J Pers Soc Psychol.

[49] Carpentier M, Van Hoye G (2020). Managing organizational attractiveness after a negative employer review: company response strategies and review consensus. Eur J Work Org Psychol.

[50] Wu L, So KKF, Xiong L, King C (2019). The impact of employee conspicuous consumption cue and physical attractiveness on consumers’ behavioral responses to service failures. IJCHM.

[51] Zhang Y, Prayag G, Song H (2021). Attribution theory and negative emotions in tourism experiences. Tour Manag Perspect.

[52] Schlosser AE (2011). Can including pros and cons increase the helpfulness and persuasiveness of online reviews? The interactive effects of ratings and arguments. J Consum Psychol.

[53] Xia L, Bechwati NN (2008). Word of Mouse: the role of cognitive personalization in online consumer reviews. J Interact Advert.

[54] Holbrook MB (2018). Beyond attitude structure: toward the informational determinants of attitude. J Mark Res.

[55] Darley WK, Smith RE (2018). Advertising claim objectivity: antecedents and effects. J Mark.

[56] Pratap Jain S, Buchanan B, Maheswaran D (2008). Comparative versus noncomparative advertising: the moderating impact of prepurchase attribute verifiability. J Consum Psychol.

[57] Cowley E (2006). Processing exaggerated advertising claims. J Bus Res.

[58] Wan Y, Zhang Y, Wang F, Yuan Y (2022). Retailer response to negative online consumer reviews: how can damaged trust be effectively repaired?. Inf Technol Manag.

[59] Piehler R, Schade M, Hanisch I, Burmann C (2019). Reacting to negative online customer reviews. J Serv Theory Pract.

[60] Bradley G, Sparks B (2012). Explanations: if, when, and how they aid service recovery. J Serv Mark.

[61] Weiner B (1985). An attributional theory of achievement motivation and emotion. Psychol Rev.

[62] Chen Z, Lurie NH (2013). Temporal contiguity and negativity bias in the impact of online word of mouth. J Mark Res.

[63] Le LH, Ha Q (2021). Effects of negative reviews and managerial responses on consumer attitude and subsequent purchase behavior: an experimental design. Comput Hum Behav.

[64] Casado-Díaz AB, Andreu L, Beckmann SC, Miller C (2018). Negative online reviews and webcare strategies in social media: effects on hotel attitude and booking intentions. Curr Issues Tour.

[65] Xu Y, Zhang Z, Law R, Zhang Z (2019). Effects of online reviews and managerial responses from a review manipulation perspective. Curr Issues Tour.

[66] Health160. 91160.com.

[67] Grewal D, Krishnan R, Baker J, Borin N (1998). The effect of store name, brand name and price discounts on consumers' evaluations and purchase intentions. J Retail.

[68] Doney PM, Cannon JP (2018). An examination of the nature of trust in buyer-seller relationships. J Mark.

[69] Han X, Wang F, Lv S, Han W (2021). Mechanism linking AR-based presentation mode and consumers’ responses: a moderated serial mediation model. J Theor Appl Electron Commer Res.

[70] Fornell C, Larcker DF (2018). Evaluating structural equation models with unobservable variables and measurement error. J Mark Res.

[71] Guetz B, Bidmon S (2022). The impact of social influence on the intention to use physician rating websites: moderated mediation analysis using a mixed methods approach. J Med Internet Res.

[72] Saleh MI (2022). Attribution theory revisited: probing the link among locus of causality theory, destination social responsibility, tourism experience types, and tourist behavior. J Travel Res.

[73] Weitzl W, Hutzinger C, Einwiller S (2018). An empirical study on how webcare mitigates complainants’ failure attributions and negative word-of-mouth. Comput Hum Behav.

[74] Swanson SR, Hsu MK (2010). The effect of recovery locus attributions and service failure severity on word-of-mouth and repurchase behaviors in the hospitality industry. J Hosp Tour Res.

[75] Bailey A (2004). Thiscompanysucks.com: the use of the Internet in negative consumer‐to‐consumer articulations. J Mark Commun.

[76] Archak N, Ghose A, Ipeirotis PG (2011). Deriving the pricing power of product features by mining consumer reviews. Manag Sci.

[77] Lee K, Koo D (2012). Effects of attribute and valence of e-WOM on message adoption: moderating roles of subjective knowledge and regulatory focus. Comput Hum Behav.

[78] Detz A, López A, Sarkar U (2013). Long-term doctor-patient relationships: patient perspective from online reviews. J Med Internet Res.

[79] Ybarra O (2002). Naive causal understanding of valenced behaviors and its implications for social information processing. Psychol Bull.

[80] Li C, Cui G, Peng L (2018). Tailoring management response to negative reviews: the effectiveness of accommodative versus defensive responses. Comput Hum Behav.

[81] Mauri AG, Minazzi R (2013). Web reviews influence on expectations and purchasing intentions of hotel potential customers. Int J Hosp Manag.

[82] Arendt F, Forrai M, Findl O (2020). Dealing with negative reviews on physician-rating websites: an experimental test of how physicians can prevent reputational damage via effective response strategies. Soc Sci Med.

