# Supplementary material for: Effect of Negative Online Reviews and Physician Responses on Health Consumers’ Choice: Experimental Study
Source: J Med Internet Res. 2024 Mar 12;26:e46713. doi: 10.2196/46713 (PMC10966444; doi:10.2196/46713)
Supplement: Multimedia Appendix 1 [file jmir_v26i1e46713_app1.docx]

1.Age

○<=24 ○25-30 ○31-35 ○36-40 ○41-45 ○>=46

2.Gender

○Male ○Female

3.Your monthly income level (after tax)

○3000 yuan and below ○3001-8000 yuan ○8001-12000 yuan ○12001 yuan and above

4. How many hours do you spend using the Internet in your work and life every day? (T stands for time)

○T ≤3h ○3<T≤5h ○5<T≤7h ○7<T≤9h ○9<T≤11h ○11<T≤13h ○T>13h

5. What is your marital status?

A Married B Unmarried C Divorced or widowed D Others_______

6. What is your working status?

○ Student ○ In employment ○Unemployed ○ Retirement ○ Freelance worker

○ Other _ _ _ _ _ _

7. What is your highest education ?

○ Junior middle school and below ○ Senior middle school

○ College or Bachelor ○ Master or Doctor

8. Have you ever used online medical service websites (WeDoctor, 91160, good doctor, etc.) to seek for physician information?

○ Yes ○ No

9. Aunt Wang is your familiar neighbor, and she has a stomachache for some days. She finds a physician on 91160, which is a widely used online medical website. You are requested to help her to decide whether the physician is a right choice. Regarding basic rating information of the physician on the website (see the picture below), report you agreement with the following statements: (1strongly disagree, 4 uncertain, 7strongly agree).


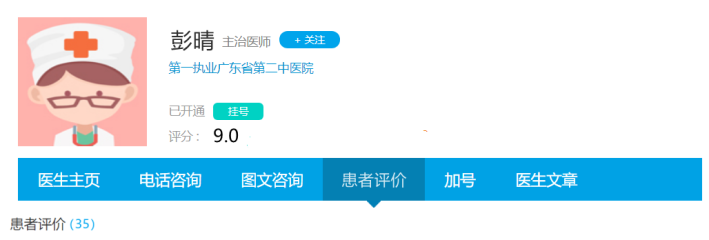


Review quantity

Title

Name

Overall Rating

Hospital

**Initial Intention**

The likelihood of considering this physician is: (very low to very high)

I consider this physician a good alternative.

My willingness to shortlist this physician is: (very low to very high)

Then, the physicians’ detailed reviews are displayed (two scenarios are presented in this appendix). Read the reviews carefully, and then report your agreement with the statement below.


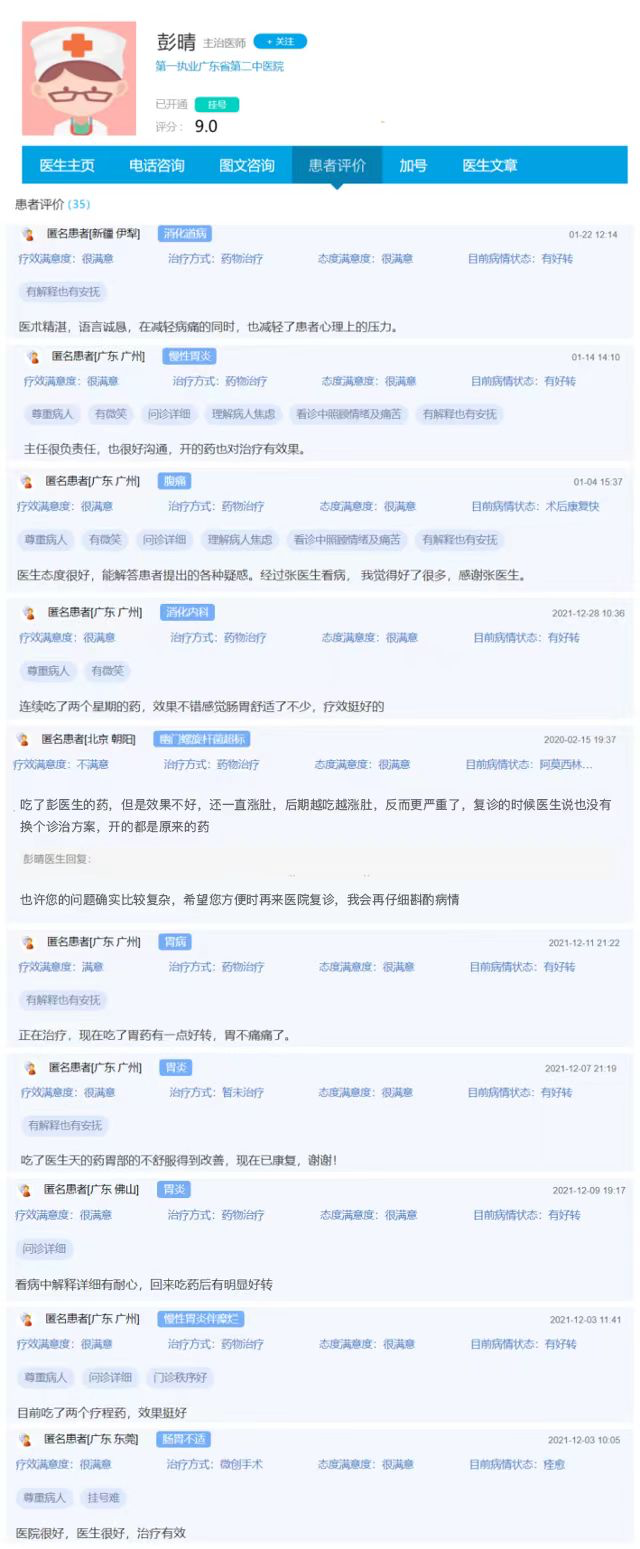


Under treatment, I have taken some stomach medication. It improved slightly. My stomach is no longer painful.

Perhaps your problem is quite complex. I hope you can come to the hospital for further consultation at your convenience, and I will carefully consider your condition

**Physician Response**

I took the medicine prescribed by Dr. Peng, but the effect was not good and my stomach continued to swell. Later on, I ate more and more, but it became even more severe. When I came back for a follow-up visit, I told the doctor. She did not change my treatment plan, and prescribed the same medicine as before.


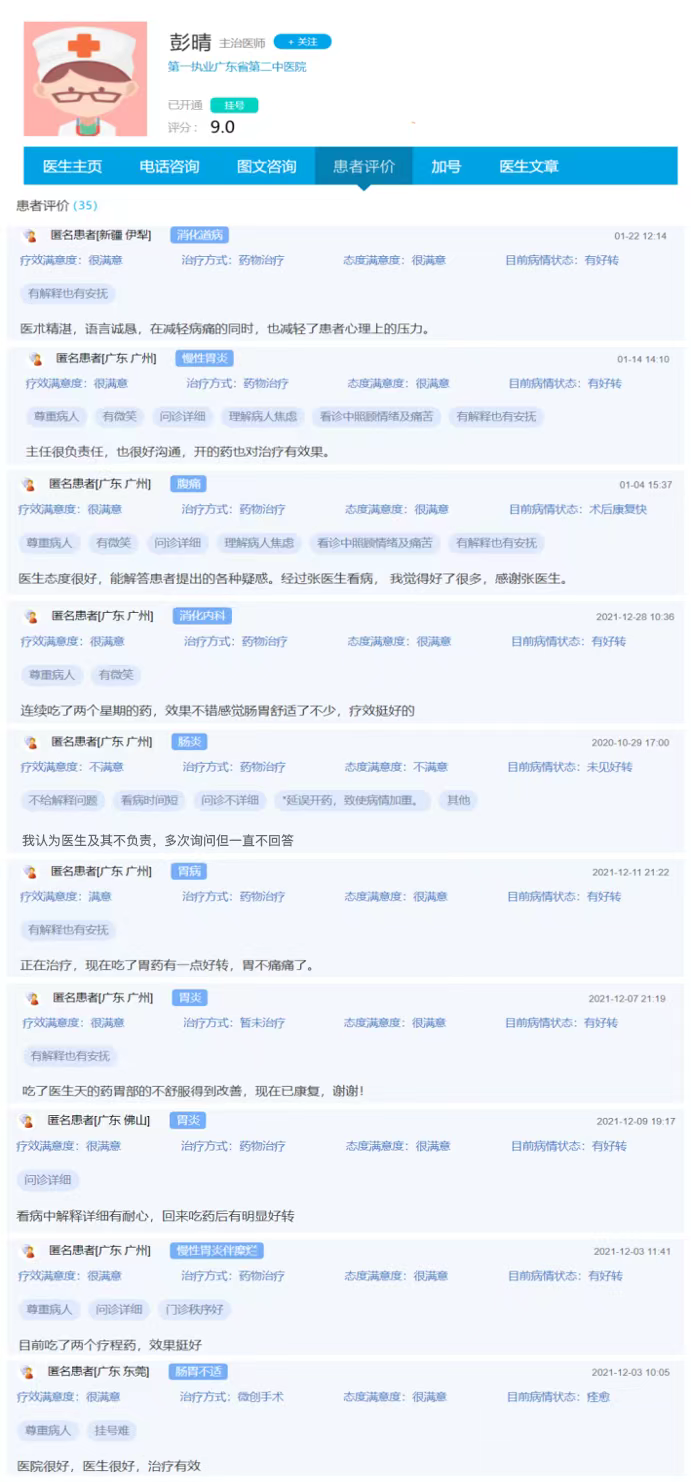


I think the doctor is extremely irresponsible. I asked multiple times but got no answer.

With exquisite medical skills and sincere attitude, she not only alleviates the pain but also alleviates the psychological pressure on patients

I have been taking the medicine for two weeks continuously, and the effect is good. I feel that my stomach and intestines are much more comfortable, and the therapeutic effect is quite good.

The proportion of negative reviews for the physician is high.

The proportion of negative reviews for the physician is low.

The negative reviews are objective facts.

The negative reviews are emotional expressions.

The physician responds to negative reviews.

The physician keeps silent on negative reviews.

The reason for the negative reviews is from the physician.

The physician is responsible for the negative reviews.

Negative reviews prove this physician is irresponsible.

I think the negative reviewers are emotional.

I think the negative reviewers are not objective enough.

I think the negative reviewers are responsible for their comments.

The likelihood of considering this physician is: (very low to very high)

I consider this physician a good alternative.

My willingness to shortlist this physician is: (very low to very high)
